# Identification of breed-specific genomic variants in Colombian Creole pig breeds by whole-genome sequencing

**DOI:** 10.1007/s11250-023-03557-9

**Published:** 2023-04-11

**Authors:** Rafael Suárez-Mesa, Roger Ros-Freixedes, Houda Laghouaouta, Ramona N. Pena, Byron Hernández-Ortiz, Iang Rondón-Barragán, Joan Estany

**Affiliations:** 1 Department of Animal Science, University of Lleida Agrotecnio-CERCA Center, 191 Rovira Roure, 25198 Lleida, Catalonia Spain; 2 Research Group in Immunobiology and Pathogenesis, Faculty of Veterinary Medicine and Zootechnics, University of Tolima, Barrio Santa Helena Parte Alta, Ibagué, Colombia; 3 Research and Innovation Group in Animal Health and Welfare Germplasm Animal Bank, Agrosavia, Bogotá 250047 Colombia

**Keywords:** Animal genetic resources, Conservation, Creole pigs, Genetic variability, *LEPR*, Whole-genome sequencing

## Abstract

Dissecting genetic variation of local breeds is important for the success of conservation. In this research, we investigated the genomic variation of Colombian Creole (CR) pigs, with a focus on the breed-specific variants in the exonic region of 34 genes with reported effects on adaptive and economic traits. Seven individuals of each of the three CR breeds (CM, Casco de Mula; SP, San Pedreño; and ZU, Zungo) were whole-genome sequenced along with 7 Iberian (IB) pigs and 7 pigs of each of the four most used cosmopolitan (CP) breeds (Duroc, Landrace × Large White, and Pietrain). Molecular variability in CR (6,451,218 variants; from 3,919,242, in SP, to 4,648,069, in CM) was comparable to that in CP, but higher than in IB. For the investigated genes, SP pigs displayed less exonic variants (178) than ZU (254), CM (263), IB (200), and the individual CP genetic types (201 to 335). Sequence variation in these genes confirmed the resemblance of CR to IB and indicates that CR pigs, particularly ZU and CM, are not exempt from selective introgression of other breeds. A total of 50 exonic variants were identified as being potentially specific to CR, including a high-impact deletion in the intron between exons 15 and 16 of the leptin receptor gene, which was only found in CM and ZU. The identification of breed-specific variants in genes related to adaptive and economical traits can bolster the understanding of the role of gene-environment interactions on local adaptation and points the way for effective breeding and conservation of CR pigs.

**Supplementary Information:**

The online version contains supplementary material available at 10.1007/s11250-023-03557-9.

## Introduction

Current pig production is based on highly cosmopolitan selected lines that are managed under intensive production systems. Still, there is a growing interest in the conservation of local breeds in favour of biodiversity (Ciobanu et al., 2001) and, in particular, as a source of adaptive variation against climate change disturbances. The three officially recognized Colombian Creole (CR) pig breeds (ZU, Zungo; CM, Casco de Mula; and SP, San Pedreño) are a good example of environmental adaptation, with pigs living in areas from around sea level to 3000 m of altitude (Ocampo-Gallego, 2019; Suárez-Mesa et al., 2021). Similar to other local breeds (Kušec et al., 2015), the census of CR pigs has been continuously declining in recent decades as intensive farming has replaced traditional production systems. Latest reports indicate that the three CR pig breeds are at high risk of extinction, with only 138 CM, 99 SP, and 128 ZU censed individuals (FAO, 2018). Currently, most of these individuals are maintained in three independent nucleus farms, one per breed, which are managed by the Colombian Agricultural Research Corporation (AGROSAVIA). In each nucleus farm, pigs are distributed in family groups and subjected to a circular mating system for maintaining genetic variability (Ocampo-Gallego, 2019). These pigs are amongst the few available individuals that can be used to investigate whether CR pigs harbour specific genetic variants that merit conservation.

Conservation of local breeds depends on their utility and prospects as a research, social, or economic resource (Barker, 1999). The Iberian pig, the most likely ancestor of CR pigs (Burgos et al., 2013), can be referred to as a model of how a local breed, previously at risk, is now abundantly used for producing premium pork products (García-Gudiño et al., 2021) and in genetic research (Crespo-Piazuelo et al., 2020). The identification of breed-specific polymorphisms in genes related to adaptive and performance traits (Bovo et al., 2020) can be a useful first approach to enhance the genetic value of local breeds (Herrero-Medrano et al., 2013; Muñoz et al, 2018). However, this has not yet been done in CR pigs. As in many other local breeds, the first attempts to genetically characterize CR local breeds were based on a small set of neutral markers such as microsatellites (Oslinger et al., 2006; Gélvez et al., 2015), but their relationship with relevant traits is not straightforward (Kirk & Freeland, 2011).

Next-generation DNA sequencing empowers geneticists to identify genetic variants at higher resolution than previously. Whole-genome sequencing has already been used in some European endangered local breeds (D’alessandro et al., 2019; Herrero-Medrano et al., 2014), but not in CR, where only a few variants associated with meat quality and fertility have been studied (Hernández et al., 2008; Pardo, 2016). In order to ensure the continuity of CR breeds, a more profound assessment of their genetic diversity is needed. In particular, CR breeds may carry breed-specific variants of genes related to adaptive and economic traits, such as those reported to affect relevant morphological, reproductive, growth, disease resilience, or meat quality traits. Thus, this research aims at identifying and characterizing the genetic variation in genes with potential effects on adaptive and economic traits in CR breeds through whole-genome sequencing.

## Material and methods

### Animals

Seven representative individuals from each of the three CR breeds (ZU, CM, and SP) were randomly sampled across available families (one per family to ensure representativeness) from the AGROSAVIA germplasm breeding nucleuses of La Libertad (Department of Meta), for CM, El Nus (Department of Antioquia), for SP, and Turipaná (Department of Córdoba), for ZU, from April to July 2019 (Suárez-Mesa et al., 2021). In addition, 7 Iberian (IB) and 21 cosmopolitan (CP) pigs (7 Duroc, 7 Pietrain and 7 Landrace × Large White) from the UdLGIM (University of Lleida) biobank (Estany et al., 2014) were also randomly sampled. The IB and CP pigs were chosen for being, respectively, the most likely ancestors of CR pigs and the current most representative transboundary genetic types. Finally, to better estimate the allele distribution across genetic types, the genotypes of 101 additional pigs from the Iberian trunk (IT; 53 IB and 48 Alentejano) and 194 CP (2 Pietrain, 151 Duroc, and 41 Landrace × Large White) for the 44 preselected markers (see below) were retrieved from either public data (Muñoz et al., 2018) or the UdLGIM biobank.

### Isolation of genomic DNA and whole-genome sequencing

Genomic DNA isolation was performed from blood samples. Briefly, blood samples were washed with TE buffer, then lysed in the presence of proteinase K, and DNA was purified by phenol:chloroform extraction, followed by ethanol precipitation. Finally, the DNA was resuspended and stored in TE buffer. The quantification and estimation of the quality and purity of the genomic DNA were done by spectrophotometer (NanoDrop N-1000, Thermo Fisher Scientific, Wilmington, USA). The integrity of the DNA was tested by electrophoresis on a 0.8% agarose gel and visualized by staining with ethidium bromide under UV illumination. Following the requirements of the National Center for Genomic Analysis (CNAG-CRG, Barcelona, Spain), all samples had a minimum concentration of 50 ng/µl. The concentration was estimated in a fluorometer (Qubit 4, Thermo Fisher Scientific).

The short-insert paired-end libraries for the whole-genome sequencing were prepared with a PCR-free protocol using the KAPA HyperPrep kit (Roche, Basel, Switzerland), with some modifications. In short, depending on the starting DNA available, 0.4 to 1.0 µg of genomic DNA was sheared on a Covaris™ LE220-Plus (Covaris, Brighton, UK) in order to reach the fragment size of ~ 400 bp. The fragmented DNA was size-selected for the fragment range of 220–550 bp with AMPure XP beads (Agencourt, Beckman Coulter, Nyon, Switzerland). The size-selected genomic DNA fragments were end-repaired and adenylated, and adaptors with unique dual indexes and unique molecular identifiers compatible with the Illumina platform (Integrated DNA Technologies, Leuven, Belgium) were ligated. The libraries were quality-controlled on an Agilent 2100 Bioanalyzer with the DNA 7500 assay (Agilent, Madrid, Spain) for size and quantified by KAPA Library Quantification Kit for Illumina platforms (Roche). The libraries were sequenced on a NovaSeq6000 (Illumina, San Diego, CA, USA) platform in paired-end mode with a read length of 2 × 151 + 17 + 8 bp following the manufacturer’s protocol for dual indexing. Image analysis, base calling, and quality scoring of the run were processed using the manufacturer’s software Real Time Analysis (RTA 3.4.4, Illumina) and followed by generation of FASTQ sequence files. A minimum of 20 Gb of sequencing data was generated per sample.

### Sequence data processing and variant discovery

The sequence reads were pre-processed using Trimmomatic (Bolger et al., 2014) to remove the adapters from the sequences DNA. The reads were aligned to the reference genome Sscrofa11.1 (GenBank accession: GCA_000003025.6) using the BWA-MEM algorithm (Li, 2013). Duplicates were marked for exclusion with Picard (http://broadinstitute.github.io/picard/). Single nucleotide polymorphisms (SNPs) and short insertions and deletions (indels) were identified with the variant caller GATK HaplotypeCaller (GATK 3.8.0) (DePristo et al., 2011; Poplin et al., 2017) using default settings. The average realized sequencing coverage was 7.9 × (SD 2.4 ×). Variant discovery with GATK HaplotypeCaller was performed separately for each individual and then the individuals in each population were jointly genotyped by extracting the variant positions from all the individuals. We retained all biallelic variants for further analyses with VCFtools (Danecek et al., 2011). Variants with minor allele frequency below 0.01 (jointly considering the sequenced individuals from all genetic types) or with a genotyping rate below 90% were removed using PLINK 1.9 software (Chang et al., 2015). This software was also used to perform a principal component analysis of genomic data to investigate population structure.

### Genetic variants in candidate genes

We preselected 44 SNPs in 34 genes with reported effects on morphological, reproductive, and response to disease-related traits (Table 1) and on growth, fatness, and meat and fat quality traits (Table 2) in pigs. The alleles for all variants are described based on the positive strand of the Sscrofa11.1 pig genome assembly. The genotypes of the sequenced pigs for these 44 SNPs were retrieved from the whole-genome sequence data, and the frequency of each allele in each breed was calculated.

**Table 1:** Investigated genetic variants in genes with reported effects on morphological, reproductive and adaptive (i.e. response to disease) traits

Variant	Gene	SSC	Position (bp)	Reference allele	Alternative allele	Trait	Reference
ACTN1_1	*ACTN1*	7	92,555,961	T	C	Fertility, piglets born alive	Wimmers et al. (2005)
ADIPOQ_1	*ADIPOQ*	13	124,643,017	G	A	Morphology, fatness	Zhang et al. (2014)
AHR_1	*AHR*	9	86,550,830	G	T	Litter size	Bosse et al. (2014)
AHR_2	*AHR*	9	86,549,936	A	C	Age at puberty	Zhang et al. (2020)
AHR_3	*AHR*	9	86,551,088	T	C	Female age at puberty	Zhu et al. (2017)
FUT1_1	*FUT1*	6	54,079,560	T	C	Diseases resistance	Wang et al. (2012)
GBP5_1	*GBP1*	4	127,301,202	G	T	Early host response to PRRS virus	Kommadath et al. (2017); Jeon et al. (2021)
HSP70_1	*HSP70*	7	23,925,510	C	A	Sperm concentration, sperm motility	Huang et al. (2002)
HSP70_2	*HSP70*	7	23,914,842	C	A	Sperm concentration, sperm motility	Huang et al. (2002)
HSP70_3	*HSP70*	7	23,914,955	T	C	Sperm concentration, sperm motility	Huang et al. (2002)
HSP70_4	*HSP70*	7	23,925,859	T	C	Sperm concentration, sperm motility	Huang et al. (2002)
KIT_1	*KIT*	8	41,488,472	C	T	Coat colour	Johansson et al. (2005); Fontanesi et al. (2010)
MC1R_1	*MC1R*	6	181,461	T	C	Coat colour	Kijas et al. (1998); Kijas et al. (2001)
MC1R_2	*MC1R*	6	181,697	A	G	Coat colour	Kijas et al. (1998); Kijas et al. (2001)
MC1R_3	*MC1R*	6	181,818	C	T	Coat colour	Kijas et al. (1998); Kijas et al. (2001)
MC1R_4	*MC1R*	6	181,825	A	G	Coat colour	Kijas et al. (1998); Kijas et al. (2001)
MC1R_5	*MC1R*	6	181,905	C	T	Coat colour	Kijas et al. (1998); Kijas et al. (2001)
MUC4_1	*MUC4*	13	134,226,654	C	G	Resistance to colibacteriosis	Schroyen et al. (2012)
NR6A1_1	*NR6A1*	1	265,347,265	A	G	Number of vertebrae	Fontanesi et al. (2014)
PPARD_1	*PPARD*	7	31,281,804	G	A	Ear size	Ren et al. (2011)
TAS2R39_1	*TAS2R39*	18	7,068,883	T	G	Growth	Ribani et al. (2017)

**Table 2:** Investigated genetic variants in genes with reported effects on growth, fatness, and meat and fat quality traits

Variant	Gene	SSC	Position (bp)	Reference allele	Alternative allele	Traits affected	Reference
ACACA_1	*ACACA*	12	3,862,4687	G	A	Carcass fatness, meat quality, fat composition	Muñoz et al. (2013)
CAPNS1_1	*CAPNS1*	6	45,514,212	A	C	Meat quality	Gandolfi et al. (2011a)
CAST_1	*CAST*	2	103,299,934	G	A	Meat quality (tenderness)	Meyers & Beever (2008)
CAST_2	*CAST*	2	103,327,456	G	A	Meat quality	Gandolfi et al. (2011b)
CTSK_1	*CTSK*	4	98,393,909	G	A	Carcass fatness, meat quality (IMF)	Fontanesi et al. (2010)
CYB5A_1	*CYB5A*	1	149,737,752	G	T	Meat quality (boar taint)	Peacock et al. (2008); Lin et al. (2005)
CYP2E1_1	*CYP2E1*	14	141,702,809	A	G	Meat quality (boar taint)	Lin et al. (2006)
FADS2_1	*FADS2*	2	9,667,336	C	T	Fat composition	Gol et al. (2018)
FASN_1	*FASN*	12	926,299	G	A	Carcass fatness, meat quality, fat composition	Muñoz et al. (2007)
FTO_1	*FTO*	6	31,460,242	A	T	Growth, carcass fatness	Dvořáková et al. (2012)
LEP_1	*LEP*	18	20,111,759	A	G	Growth, carcass fatness, feed intake	Kennes et al. (2001)
LEPR_1	*LEPR*	6	146,829,589	G	A	Growth, fatness, meat quality (IMF), feed intake	Óvilo et al. (2010); Óvilo et al. (2005)
MC4R_1	*MC4R*	1	160,773,437	G	A	Feed intake, growth, carcass fatness	Kim et al. (2000)
MSTN_1	*MSTN*	15	94,629,248	C	T	Growth, carcass fatness	Tu et al. (2014)
MTTP_1	*MTTP*	8	120,821,998	G	A	Meat quality, fat composition	Estellé et al. (2009)
PCK1_1	*PCK1*	17	57,932,233	A	C	Growth, carcass fatness, meat quality (IMF)	Latorre et al. (2016)
PHKG1_1	*PHKG1*	3	16,830,320	C	A	Carcass fatness, meat quality (pH)	Ma et al. (2014)
PLIN1_1	*PLIN1*	7	55,250,707	C	T	Growth	Gol et al. (2016)
PLIN2_1	*PLIN2*	1	203,694,497	A	G	Growth, carcass fatness	Gol et al. (2016)
PPARGC1A_1	*PPARGC1A*	8	17,867,068	A	T	Meat quality	Gandolfi et al. (2011a)
PRKAG3_1	*PRKAG3*	15	120,863,537	C	T	Meat quality (pH)	Ciobanu et al. (2001); Milan et al. (2000)
RYR1_1	*RYR1*	6	47,357,966	T	C	Growth, carcass fatness, meat quality (pH)	Roberts et al. (2001); Fujii et al. (1991)
SCD_1	*SCD*	14	111,461,751	C	T	Fat composition	Estany et al. (2014)

In addition, we also called all the variants along the exonic regions of the genes in Tables 1 and 2 that are annotated in the Sscrofa11.1 assembly of the pig genome (27 of 34 genes). These variants were grouped according to whether they were common (i.e. called in all genetic types) or breed-specific (i.e. called in only one breed or group of breeds, in particular to CR) and to their predicted impact using the Ensembl Variant Effect Predictor (VEP) tool (McLaren et al., 2016).

## Results

### *Number of the called variants*

**Fig. 1:**
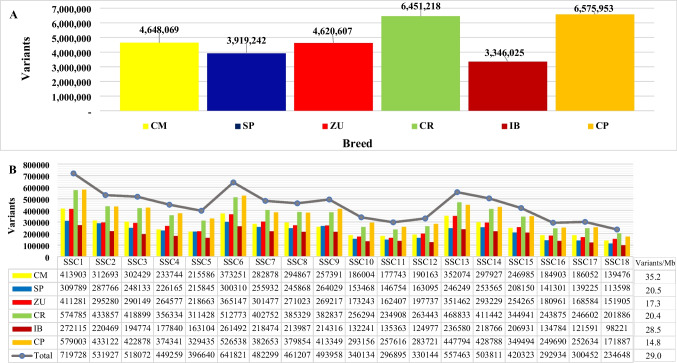
Total number of the so-called variants by breed (**A**) and chromosome (**B**) in Colombian Creole (CR, *n* = 21, with CM, Casco de Mula; ZU, Zungo; SP, San Pedreño), Iberian (IB, *n* = 7), and cosmopolitan (CP, *n* = 21) pigs. A number of the so-called variants in the individual CP genetic types were 3,723,941 in Duroc, 5,068,938 in Landrace × Large White, and 4,391,019 in Pietrain

**Fig. 2:**
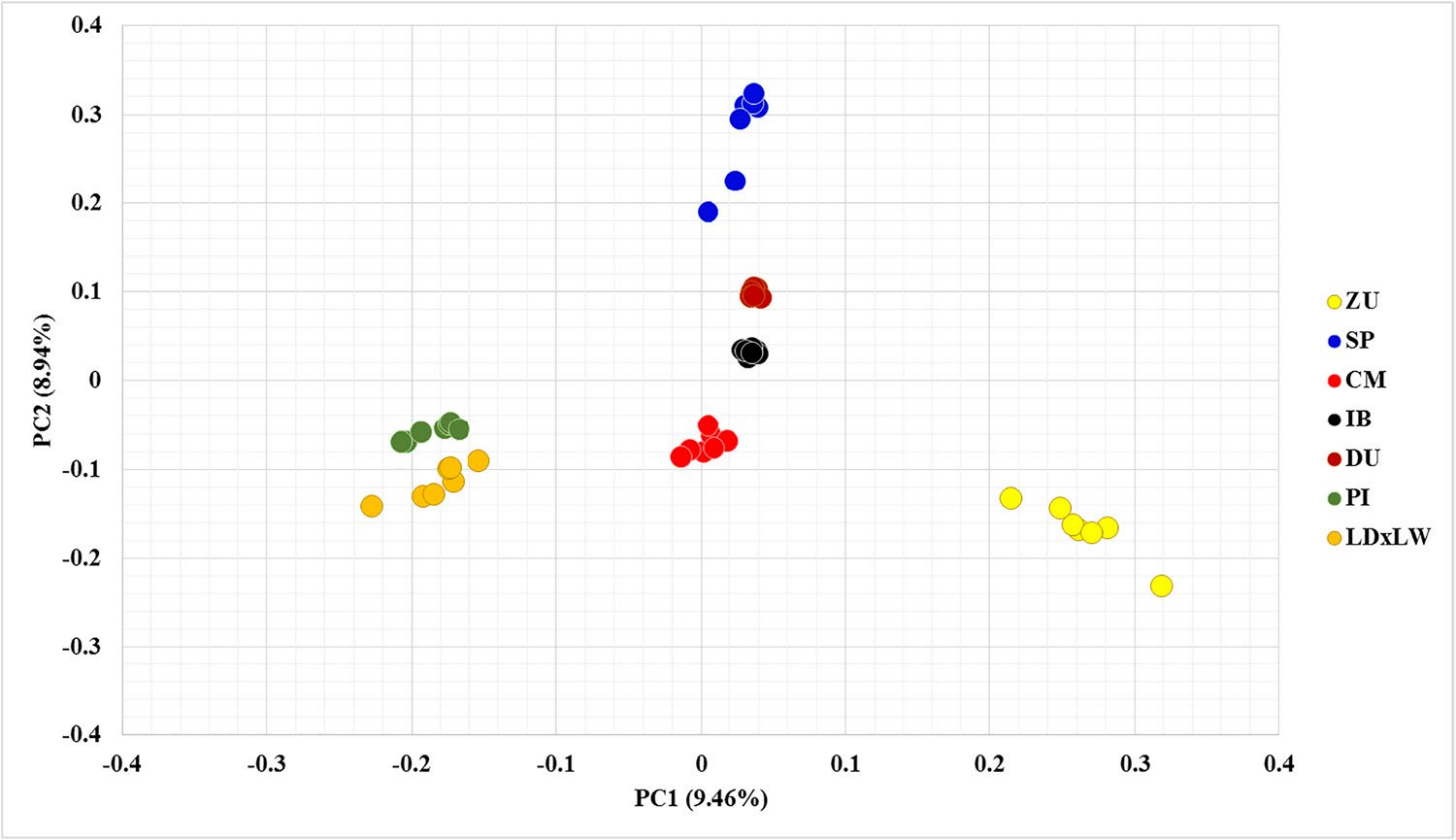
Scatter plot of the two first principal components (PC1 and PC2, in parenthesis the variance explained) for genome sequence in Colombian Creole (CM, Casco de Mula; SP, San Pedreño; and ZU, Zungo), Iberian (IB) and Cosmopolitan pig breeds (PI: Pietrain; DU: Duroc; and LDxLW: Landrace × Large White). Each circle represents a subject

We present here the first whole-genome sequence data of CR pig breeds (Fig. 1A). The total number of genetic variants called across all sequenced types was 7,971,714. Of them, 6,451,218 were called in CR, which was in line with the number of variants found in CP (6,575,953). The number of variants in each CR breed ranged from 3,919,242, in SP, to 4,648,069, in CM, which was higher than in IB (3,346,025) and similar to CP types (from 3,723,941, in Duroc, to 5,068,938, in Landrace × Large White). As expected, the number of variants per chromosome increased with chromosome size. The CR pigs presented a higher average variant density (20.4 variants/Mb) than the CP pigs (14.8 variants/Mb) (Fig. 1B). Despite the limited number of pigs per breed, sequence data revealed a sensible population structure. As can be seen from the outcome of the principal component analysis of the so-called variants, pigs of the same breed get clustered together (Fig. 2). The first principal component clearly differentiated white types (Pietrain and Landrace × Large White) from ZU, while the second principal component distinguished the other four breeds, particularly CM from SP.

### Allele frequency of preselected variants from candidate genes

The frequency of the alternative allele (as annotated in the reference genome Sscrofa11.1) for each of the 44 SNPs in Tables 1 and 2 is given in Tables 3 and 4, respectively. The CR pigs showed relatively high frequencies for some of the alternative alleles. As a result, the number of variants that were fixed (i.e. only one of the two alleles of the variant was present in the sample of pigs used for this study) was lower in CR (6) than in IT (13), although higher than in CP (4). There was also variability in the alternative allele frequency of the preselected variants across CR breeds. Twenty-eight of them were fixed in at least one CR breed, with SP having the largest number of fixed variants (21), ZU the fewest (10), while CM had an intermediate number (16). Ten of the 13 variants (from 8 of 34 genes) that were fixed in IT were also fixed in SP, including AHR_2, AHR_3**,** LEPR_1, MC4R_1, and SCD_1, but only 6 (from 5 genes) and 3 (from 3 genes) in ZU and CM, respectively. Interestingly, the maximum differences in allele frequency across genetic types occurred for LEPR_1, where the frequency of the alternative allele ranged from values lower than 0.3, in CP, CM, and ZU, to 1.00, in IT and SP, and for *AHR* polymorphisms, where the frequency of the alternative allele was much higher in CR and IT pigs (0.71–1.00) than in CP pigs (0.32–0.36). The other three fixed variants in IT (CAST_2, PLIN*2*_1, and GBP5_1) segregated in all three CR breeds, except for PLIN2_1 in ZU, for which the same allele as in IT was fixed. In general, the alleles fixed in CR segregated at a very high frequency in IT.

**Table 3:** Frequency of the alternative allele in the investigated variants for morphological, reproductive and adaptive (i.e. response to disease) traits in Colombian Creole, Iberian trunk and cosmopolitan pig breeds

Variant^2^	Alternative allele	Breed^1^					
		Colombian Creole					
		CM *n* = 7	SP *n* = 7	ZU *n* = 7	CR *n* = 21	IT *n* = 108	CP *n* = 215
ACTN1_1	T	0.29	0.79	0.33	0.47	0.58	0.41
ADIPOQ_1	A	0.21	0.57	0.21	0.33	0.03	0.13
AHR_1	T	0.75	1.00	0.79	0.85	0.94	0.33
AHR_2	C	0.71	1.00	0.86	0.86	1.00	0.36
AHR_3	C	0.79	1.00	0.86	0.88	1.00	0.32
FUT1_1	C	0.50	0.36	0.93	0.60	0.94	0.75
GBP5_1	T	0.07	0.36	0.64	0.36	0.00	0.19
HSP70_1	A	0.43	0.21	0.00	0.21	0.60	0.38
HSP70_2	C	0.00	0.50	0.50	0.33	0.75	0.63
HSP70_3	A	0.00	0.00	0.00	0.00	0.17	0.01
HSP70_4	C	1.00	1.00	0.50	0.83	0.88	0.76
KIT_1	T	0.07	0.00	0.00	0.02	0.00	0.00
MC1R_1	T	0.00	0.14	0.29	0.14	0.01	0.00
MC1R_2	C	1.00	1.00	1.00	1.00	1.00	0.67
MC1R_3	G	1.00	1.00	1.00	1.00	1.00	0.67
MC1R_4	T	1.00	0.86	0.79	0.88	0.76	0.67
MC1R_5	G	0.00	0.14	0.21	0.12	0.01	0.00
MUC4_1	G	0.00	0.00	0.07	0.02	0.03	0.29
NR6A1_1	G	0.07	0.00	0.00	0.02	0.14	0.00
PPARD_1	A	0.00	0.00	0.50	0.17	0.02	0.01
TAS2R39_1	G	1.00	1.00	1.00	1.00	0.96	0.79

Bold type indicates allele fixation. ^1^Colombian Creole breeds (*CM*, Casco de Mula; *SP*, San Pedreño; *ZU*, Zungo; *CR*, all three Colombian Creole breeds); *IT*, Iberian trunk pigs (60 Iberian and 48 Alentejano); and *CP*, cosmopolitan breeds (9 Pietrain; 158 Duroc; and 48 Landrace × Large White). ^2^See Table 1 for variant description

**Table 4:** Frequency of the alternative allele in the investigated variants for growth, fatness and meat and fat quality traits in Colombian Creole, Iberian trunk and cosmopolitan pig breeds

Variant ^2^	Alternative allele	Breed^1^					
		Colombian Creole					
		CM *n* = 7	SP *n* = 7	ZU *n* = 7	CR *n* = 21	IT *n* = 108	CP *n* = 215
ACACA_1	A	0.57	0.50	0.79	0.62	0.57	0.36
CAPNS1_1	C	0.71	0.50	0.93	0.71	0.48	0.52
CAST_1	A	0.21	0.50	0.43	0.38	0.83	0.35
CAST_2	A	0.21	0.14	0.43	0.26	0.00	0.35
CTSK_1	A	0.00	0.00	0.00	0.00	0.00	0.08
CYB5A_1	T	0.00	0.00	0.43	0.14	0.48	0.07
CYP2E1_1	G	0.57	0.07	0.79	0.48	0.79	0.40
FADS2_1	T	1.00	0.36	0.93	0.76	0.92	0.55
FASN_1	A	0.50	1.00	0.29	0.60	0.75	0.73
FTO_1	T	0.50	0.36	0.21	0.36	0.67	0.37
LEP_1	G	0.93	0.64	0.29	0.62	0.20	0.89
LEPR_1	A	0.21	1.00	0.29	0.50	1.00	0.23
MC4R_1	A	0.79	0.00	0.07	0.29	0.00	0.26
MSTN_1	T	0.50	0.50	0.71	0.57	0.88	0.54
MTTP_1	A	0.36	0.64	0.93	0.64	0.33	0.43
PCK1_1	C	0.36	0.00	0.43	0.26	0.03	0.55
PHKG1_1	A	0.00	0.00	0.14	0.05	0.02	0.10
PLIN1_1	T	0.93	0.64	0.79	0.79	0.59	0.44
PLIN2_1	G	0.79	0.21	1.00	0.67	1.00	0.81
PPARGC1A_1	T	0.29	0.64	0.79	0.57	0.14	0.64
PRKAG3_1	T	0.07	0.29	0.33	0.23	0.67	0.25
RYR1_1	C	1.00	1.00	1.00	1.00	1.00	0.83
SCD_1	T	0.57	1.00	0.93	0.83	1.00	0.80

Bold type indicates allele fixation. ^1^Colombian Creole breeds (*CM*, Casco de Mula; *SP*, San Pedreño; *ZU*, Zungo; *CR*, all three Colombian Creole breeds); *IT*, Iberian trunk pigs (60 Iberian and 48 Alentejano); and *CP*, cosmopolitan breeds (9 Pietrain; 158 Duroc; and 48 Landrace × Large White). ^2^See Table 2 for variant description

### Additional variants in preselected candidate genes

**Fig. 3:**
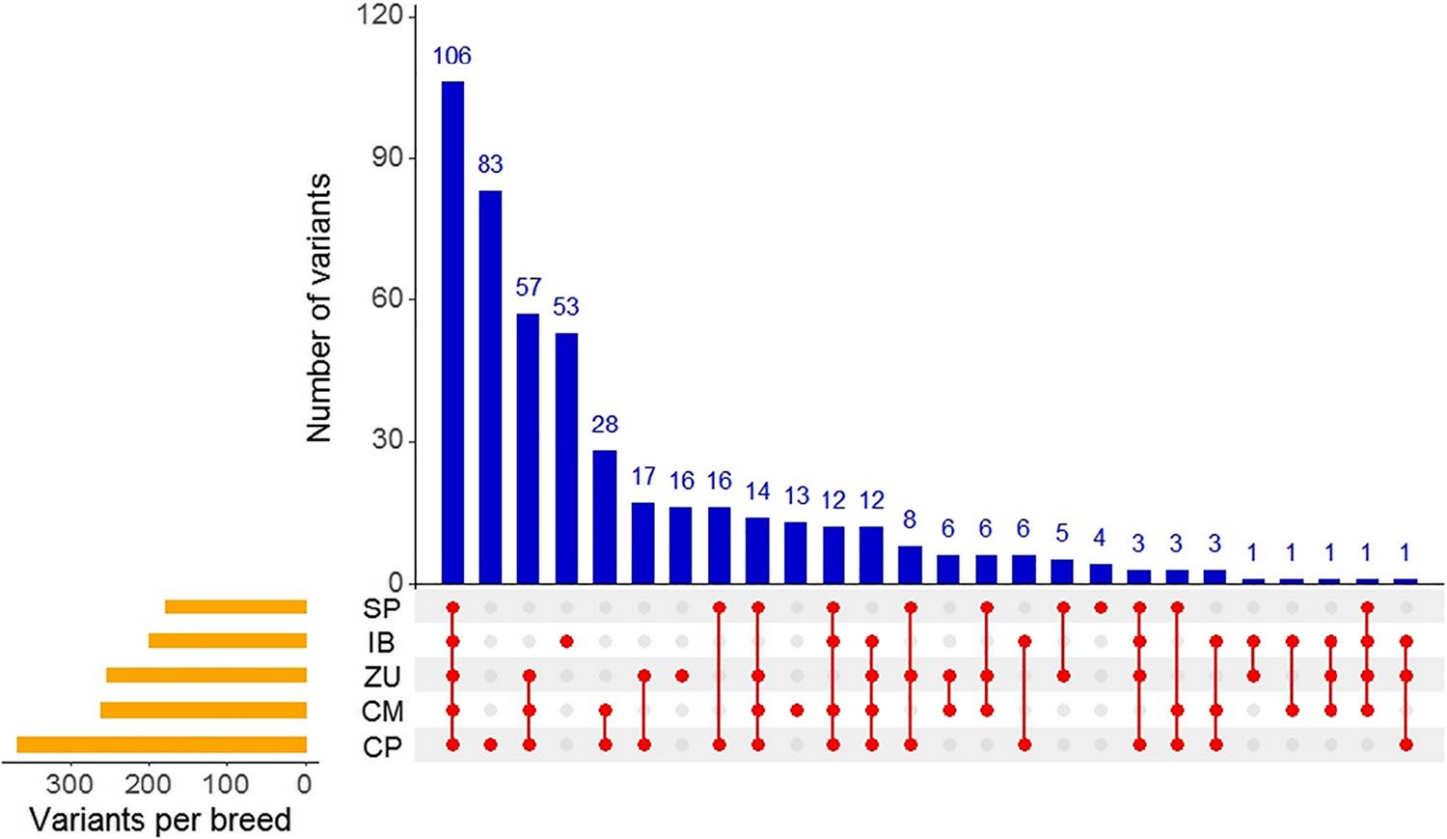
UpSet plot of the variants found in the investigated genes (Table 5). Set size is the total number of variants in each breed (CM, Casco de Mula; ZU, Zungo; SP, San Pedreño; IB, Iberian; and CP, Cosmopolitan breeds). Interaction size represents the number of variants in the intersections of the breeds as indicated by the black dots below the bars

In annotated genes (Table 5), exonic sequence variation in CR (334 variants; Supporting Table S1) was greater than in IB (200 variants) but lower than in CP (369 variants). The SP pigs had less exonic variants (178) than the two other CR (254, for ZU, and 263, for CM), IB (200), and CP breeds (201, for Pietrain, to 335, for Landrace × Large White). A total of 106 of these variants were common to all breeds, while 50 of them were specific to CR (Fig. 3). As compared to CR, the number of specific variants was similar in IB (53 variants) and higher in CP (83 variants). However, CR breeds shared less variants with IB that were not called in CP (4) than variants with CP that were not called in IB (143). Variants were categorized according to their predicted impact over mRNA transcription and protein translation and functionality (Table 6). In CR, ZU had the highest number of breed-specific variants (16), of low (11) or moderate (5) predicted impact. These variants were located in the *ACTN1*, *FADS2*, *FTO*, *FUT1*, *PHKG1*, *PLIN2*, *PRKAG3*, and *TAS2R39* genes. The breed-specific variants found in CM (13) were in another set of genes (*ADIPOQ*, *CAPNS1*, *CAST*, *FTO*, *KIT*, *LEPR*, and *PPARGC1A*) and were of low (9) or moderate (4) predicted impact. The SP breed presented only 4 breed-specific variants, 3 in the *ACACA* gene and 1 in the *FADS2* gene, all of them of low predicted impact.

**Table 5:** Number of identified exonic variants in the investigated genes by breed ^1^ and predicted impact ^2^

	Breed																							
	Colombian Creole																							
Gene^3^	CM, *n* = 7				SP, *n* = 7				ZU, *n* = 7				CR, *n* = 21				IB, *n* = 7				CP, *n* = 21			
	H	M	L	T	H	M	L	T	H	M	L	T	H	M	L	T	H	M	L	T	H	M	L	T
*ACACA*			11	11			20	20							20	20	1	2	31	34			32	32
*ACTN1*	3	2	19	24			13	13	3	4	22	29	3	4	23	30	2		19	21	3	4	21	28
*ADIPOQ*	1	2	4	7	2	1	2	5	2	1	3	6	2	2	5	9	1	1	2	4	2	2	4	8
*AHR*		11	8	19		9	8	17		9	8	17		11	8	19		9	8	17		11	7	18
*CAPNS1*			2	2			1	1			2	2			3	3			1	1			2	2
*CAST*		7	8	15		5	7	12		5	7	12		7	8	15		2	2	4		11	13	24
*CYP2E1*			2	2			1	1			2	2			2	2			2	2			2	2
*FADS2*	1	8	10	19	1	12	13	26	1	11	14	26	1	13	18	32	1	8	10	19	1	14	17	32
*FASN*	2	6	16	24	2	3	6	11	2	7	15	24	2	7	16	25	2	4	6	12	2	7	20	29
*FTO*			6	6			2	2			3	3			7	7			8	8			2	2
*FUT1*	1	1	1	3	1	1		2	1	2	1	4	1	2	1	4	1	2	1	4	2	3	1	6
*KIT*	1	4	20	25	1	2	10	13	1	3	19	23	1	4	20	25	1	4	12	17	2	4	12	18
*LEP*			1	1			1	1							1	1						1	1	2
*LEPR*	1	7	7	15		1	2	3	1	4	3	8	1	8	8	17		2	2	4		8	12	20
*MC1R*	1	3		4		5	3	8		5	3	8	1	5	3	9	1	3		4	1	4		5
*MC4R*		1	1	2						1		1		1	1	2						2	1	3
*MTTP*		1	10	11		2	3	5		1	9	10		2	13	15		4	7	11		3	18	21
*NR6A1*		1		1						1		1		2		2		1		1		1		1
*PCK1*	1	9	7	17	2	1	2	5	2	9	12	23	2	10	12	24	2	2	3	7	3	16	15	34
*PHKG1*		1	3	4			2	2			6	6		1	6	7		1	6	7		1	6	7
*PLIN1*	1		3	4	1	1	3	5	1	1	3	5	1	1	3	5	1		3	4	1	2	3	6
*PLIN2*		2	8	10						1	9	10		2	11	13		1		1		4	10	14
*PPARD*			3	3			2	2		1	2	3		1	3	4		1	2	3		1	3	4
*PPARGC1A*		3	2	5		2	3	5		1	2	3		3	4	7		1	1	2		3	4	7
*PRKAG3*	1	5	7	13	2	4	2	8	2	5	3	10	2	7	7	16	1	3	2	6	2	7	10	19
*SCD*			2	2			2	2			2	2			2	2			2	2			3	3
*TAS2R39*	1	9	4	14		8	1	9	1	10	5	16	1	13	5	19		4	1	5	1	15	6	22
Total	15	83	165	263	12	57	109	178	17	82	155	254	18	106	210	334	14	55	131	200	20	124	225	369^4^

Bold type indicates totals. ^1^Colombian Creole breeds (*CM*, Casco de Mula; *SP*, San Pedreño; *ZU*, Zungo; *CR*, all three Colombian Creole breeds); *IB*, Iberian; and *CP*, Cosmopolitan breeds (7 Pietrain, 7 Duroc, and 7 Landrace × Large White). ^2^Predicted impact of the variants (*H*, high; *M*, moderate; and *L*, low) using the Ensembl Variant Effect Predictor (VEP) tool; *T*, total number of variants. ^3^See Tables 1 and 2 for gene description. ^4^Duroc: 223 exonic variants in total; Large White × Landrace: 335; Pietrain: 201

**Table 6:** Number of exonic variants by breed and predicted impact over mRNA transcription and translation

	Breed^1^					
Predicted impact^2^	Colombian Creole					
	CM *n* = 7	SP *n* = 7	ZU *n* = 7	CR *n* = 21	IB *n* = 7	CP *n* = 21
High	15	12	17	18	14	20
Frameshift indel	11	10	12	13	12	14
Splice acceptor	1	1	1	1	1	2
Splice donor	2	-	2	2	1	2
Start lost	1	-	1	1	-	1
Stop lost	-	1	1	1	-	1
Moderate	83	57	82	106	55	124
In-frame deletion	3	2	3	3	2	4
Missense variant	80	55	79	103	53	120
Low	165	109	155	210	131	225
Splice region	37	21	33	45	18	47
Synonymous	128	88	122	165	113	178
Total	263	178	254	334	200	369^3^

Bold type indicates totals. ^1^Colombian Creole breeds (*CM*, Casco de Mula; *SP*, San Pedreño; *ZU*, Zungo; *CR*, all three Colombian Creole breeds); *IB*, Iberian; and *CP*, cosmopolitan breeds (7 Pietrain; 7 Duroc; and 7 Landrace × Large White). ^2^Predicted impact using the Ensembl Variant Effect Predictor (VEP). ^3^Duroc: 223 exonic variants in total; Landrace × Large White: 335; Pietrain: 201

In total, there were 18 variants with high predicted impact in CR (Supporting Table S1). Nine of them were shared amongst CR breeds and all but one (in *MC1R* and segregating in CM) were in ZU. Six genes (*ACTN1*, *ADIPOQ*, *LEPR*, *PCK1*, *PRKAG3*, and *TAS2R39)* harboured the other 8 high-impact variants. The three high-impact variants in *ACTN1* were not observed in SP and only one of the two that were identified in *PCK1*, *PRKAG3* and *ADIPOQ* were observed in CM. The high-impact variants in *LEPR* and *TAS2R39* were found in CM and ZU but not in SP. Of all high-impact variants, only the splice-donor polymorphism located in the *LEPR* gene was specific to CR. High-impact variants were mostly frameshift indels (Table 6).

## Discussion

The few analyses of genetic variation carried out so far in CR pigs were limited to a small set of neutral markers (Vargas et al., 2016). Whole-genome sequencing provides a more comprehensive resolution of the genetic variation within and across populations across all genomic regions (Ros-Freixedes et al., 2022). Here, we focused on a set of 34 candidate genes with reported effects on adaptive or economic traits (Tables 1 and 2). Six of the preselected variants in these genes were not observed in CR (CTSK_1, HSP70_3, MC1R_2, MC1R_3, RYR1_1, and TAS2R39_1), and seven were only seen in one of the CR breeds (CYB5A_1, HSP70_4, KIT_1, MUC4_1, NR6A1_1, PHKG1_1, and PPARD_1). The variants in the *MC1R*, *NR6A1*, *PPARD*, and *TAS2R39* genes are missense mutations that might have been selected for environmental adaptation.

The *MC1R* gene has a great impact in the determination of coat colour due to its key role regulating the synthesis of eumelanin (black/brown) and phaeomelanin (yellow/red) in the melanocytes (Barsh, 1996; Fang et al., 2009). At least six haplotypes, tagged by 5 SNPs (MC1R_1 to MC1R_5, Table 1) and one deletion (g.182126CC > *), have been described in this gene (Muñoz et al., 2018). In CR, we only found three of these six haplotypes (Supporting Table S2), the so-called MC1R*2 (GCGCA**), MC1R*3 (GCATG**), and MC1R*6 (GCATGCC), all of which are associated with black coat or spotting. The predominance of MC1R*3 in SP (frequency of 85.7%) and in ZU (frequency of 71.4%) is consistent with IB (likely, Lampiño) origin (Ocampo-Gallego and Abuabara-Pérez, 2021), since this haplotype is fixed in old black-coated and hairless IB strains such as Lampiño (Alves et al., 2007; Fernández et al., 2004). However, MC1R*3 was residual in CM, where MC1R*6 was the predominant haplotype (frequency of 85.7%), as happens in current commercial IB strains (Muñoz et al., 2018). The presence of the MC1R*2 in SP (frequency of 14.3%) and ZU (frequency 21.4%), which has been previously detected in Large Black, provides evidence of introgression of black alleles from Asian origin into these two breeds. Likewise, CM does not seem to be completely free of introgression from transboundary breeds, as indicates the presence of the T allele in the KIT_1 variant (belted phenotype), which is absent in IT breeds (Muñoz et al., 2018). The absence of the MC1R*4 (ATGTG**) haplotype in CR pigs indicates that they have not been crossbred with Duroc.

The *TAS2R39* gene is a member of the bitter-taste receptor family that has been related to fatness (Ribani et al., 2017). In agreement with findings in European local breeds (Muñoz et al., 2018), the G allele at TAS2R39_1 is fixed in the three CR breeds, thereby suggesting a selective pressure towards defensive bitter taste. The A allele at NR6A1_1 was fixed in SP and ZU but not in CM. This allele increases the vertebrae number in pigs, resulting in longer carcasses (Mikawa et al., 2007). This could imply that CM pigs could have been less intensively selected for body size than SP and ZU. On the other hand, PPARD_1, as well as CBY5A_1 and HSP70_4, only segregated in ZU and at intermediate frequencies. Since the ZU pigs are found in the Atlantic coastal area, where the weather is especially hot, it is worth exploring whether these three variants might be related to heat resistance, as it has been described before. For instance, the missense mutation PPARD_1 (A allele; Table 1) increases ear size in pigs (Ren et al., 2011), with implications on skin homeostasis and fat deposition. The A allele is found in Asian but not in European breeds. The fact that the A allele segregated in ZU at a frequency of 50% adds evidence of Asian introgression into this breed, which, on the other hand, is characterized by having large and droppy ears (FAO, 1992). The T allele at CYB5A_1 has been associated with low fat and androstenone levels (Peacock et al., 2008; Lin et al., 2005). While this may be desirable to reduce the risk of boar taint in carcasses from entire ZU males, it may jeopardize reproduction success. The *HSP70* variants have also been related to male reproduction. In particular, the T allele at HSP70_4 has been associated to larger ejaculates and semen quality. In a previous research we showed that CR boars produced less normal and motile sperm per ejaculate than CP boars (Suárez-Mesa et al., 2021). Allele frequency patterns of *HSP70* variants across breeds do not provide further evidence for an association of these markers with male fertility.

The rest of variants that appeared as fixed in a single CR breed were mostly found in SP. These variants were also either fixed or at very high frequency in IT (AHR_1 to AHR_3, LEPR_1, MC4R_1, PCK1_1, and SCD_1). The fatty nature of these breeds is consistent with the presence of the A allele at LEPR_1 (Table 2), which has been documented to increase feed intake and fatness and to impair reproductive and maternal abilities (Ros-Freixedes et al., 2016; Solé et al., 2021). This allele co-segregated with the T allele at AHR_1, which has a negative impact on prolificacy (Bosse et al., 2014). The joint presence of these two fixed alleles in SP can compromise the reproductive outcome of this breed. Given the sample size per breed, no clear-cut pattern can be inferred from the allele frequency of MC4R_1, PCK1_1, and SCD_1 across breeds, except that, amongst CR, SP was the closest to IT and CM the most differentiated. The CM pigs showed higher frequencies of the alleles associated with increased fatness (A allele at MC4R_1; Kim et al., 2004; and C allele at PCK1_1; Latorre et al., 2016) and saturated fatty acid abundance (C allele at SCD_1; Estany et al., 2014).

A total of 27 of the studied genes are annotated in the Sscrofa11.1 assembly of the pig genome. Therefore, in a second step, we went further to search for new variants into the coding region of these genes using whole-genome sequence data. In CR, we found 18 variants of high impact on mRNA sequence and protein translation. Only one of them was specific to CR. This is a splice-donor variant in *LEPR* that consists of a 7-bp deletion extending upstream on intron between exons 15 and 16 (LEPR_2: SSC6:146,829,573–146,829,580 bp) that affects the three transcripts of the gene. This deletion was only observed in CM and ZU (frequency of 7.1% and 28.6%, respectively) and was fully linked to the G (non-fatty) allele in LEPR_1*,* but not vice versa. Since these two variants are separated by only 9 bp, we can hypothesize that the 7-bp deletion appeared later from a haplotype with the G allele at LEPR_1. No homozygous individuals for the deletion allele were found, even though the probability of sampling at least one in ZU was around 45%. Apart from LEPR_1, other seven variants of moderate impact were detected in CR for *LEPR* (Table 5; Supporting Table S3). In line with results for LEPR_2, SP pigs did not show the alternative allele in these variants (LEPR_3: SSC6:146,831,558 bp, frequency of 57.1%, only in CM; LEPR_4: SSC6:146,838,276 bp, frequency of 7.1%, in CM, and 50.0%, in ZU; LEPR_5: SSC6:146,838,380 bp, frequency of 21.4.0%, only in ZU; LEPR_6: SSC6:146,847,237 bp, frequency of 21.4%, only in CM; LEPR_7: SSC6:146,861,093 bp, frequency of 42.9%, only in CM; LEPR_8: SSC6:146,861,094 bp, frequency of 42.9%, only in CM; and LEPR_9: SSC6:146,861,105 bp, frequency of 64.3%, in CM, and 14.3%, in ZU). Interestingly, for LEPR_1 to LEPR _9, the same allele was fixed in SP and IB. However, considering all LEPR variants in Table 5, we can infer that, in SP, all of these variants reside in a single haplotype of 35,019 bp (from SSC6:146,826,086 bp to SSC6:146,861,105 bp), while, in IB, they are inherited in two haplotypes due to a specific in-between missense variant at SSC6:146,830,356 bp (frequency of 54.2%). More detailed studies are needed to decipher the connection between *LEPR* variants and their effects on phenotypes. Nevertheless, findings so far provide further evidence on the IB origin of SP and give clues about the role that *LEPR*, as a key element of the endocrine control of energy balance (Friedman, 2019), may have played in the adaptation of CR breeds to different geographical locations and dietary regimes.

Besides those in *LEPR*, we identified 12 more missense mutations. Three of them were present in more than one CR breed and affected genes involved in coat colour (*KIT*, in the three CR breeds, and *MC1R*, in SP and ZU). The remaining 9 were observed in CM and ZU (*FASN*) or only in CM (*ADIPOQ*, *CAST*, and *PPARGC1A*) or ZU (*FADS2*, *FUT1*, *ACTN1*, and *PRKAG3*). No breed-specific missense mutations were found in SP, a result that would confirm that molecular variability is lower in this breed as compared to CM and ZU. As a whole, our findings support that genome-wide characterization is a useful tool to identify patterns of genetic variation between and within CR breeds.

## Conclusions

This is the first study that characterizes genetic variation at the whole-genome sequence level in CR pigs. The molecular variability of the three CR breeds is comparable to CP breeds, although higher in ZU and CM than in SP. Despite the limited sample size per breed, the sequence variation of the 34 investigated genes would confirm the relationship between CR and IB pigs, but also that they are not exempt from selective introgression of transboundary breeds, particularly ZU and CM. Differential allele distribution across breeds provides evidence to understand the genetic makeup of the CR breeds for body size, fatness, skin colour, ear size, and boar taint. The identification of 50 sequence variants that are potentially specific to CR points the way forward for further research and adds new data to inform breed development and conservation decisions. The discovery of a novel variant of *LEPR* in CM and ZU can give new clues on the role of *LEPR-*environment interactions on local adaptation. Our findings reinforce the need for ad hoc phenotyping schemes in order to experimentally validate in silico predictions of the impact of such variants on adaptive and economical traits and to develop effective breeding and conservation programmes for CR breeds.

## Supplementary Information

Below is the link to the electronic supplementary material.

Supplementary Table S1 (XLSX 28.6 KB)

Supplementary Table S2 (DOCX 19.1 KB)

Supplementary Table S3 (DOCX 18.1 KB)

## Data Availability

Please contact author for data requests.
