# Supplementary material for: Identification of breed-specific genomic variants in Colombian Creole pig breeds by whole-genome sequencing
Source: Trop Anim Health Prod. 2023 Apr 11;55(3):154. doi: 10.1007/s11250-023-03557-9 (PMC10089996; doi:10.1007/s11250-023-03557-9)
Supplement: Supplementary file 2 — Supplementary Table S2 (DOCX 19.1 KB) [file 11250_2023_3557_MOESM2_ESM.docx]

**Supporting Table S2.** Individual genotypes of sequenced Colombian Creole pigs for the melanocortin 1 receptor (*MC1R*) gene variants

| **Breed (pig no.)** | **Genotype ^1^** | | | | | | **Expected haplotype** |
| --- | --- | --- | --- | --- | --- | --- | --- |
|  | **g.181461G>A** | **g.181697C>T** | **g.181818G>A** | **g.181825T>C** | **g. 181905G>A** | **g.182120CC>*** |  |
| CM (1) | GG | CC | AA | TT | GG | CC/CC | MC1R*6/MC1R*6 |
| CM (2) | GG | CC | AA | TT | GG | CC/CC | MC1R*6/MC1R*6 |
| CM (3) | GG | CC | AA | TT | GG | **/CC | MC1R*3/MC1R*6 |
| CM (4) | GG | CC | AA | TT | GG | CC/CC | MC1R*6/MC1R*6 |
| CM (5) | GG | CC | AA | TT | GG | **/CC | MC1R*3/MC1R*6 |
| CM (6) | GG | CC | AA | TT | GG | CC/CC | MC1R*6/MC1R*6 |
| CM (7) | GG | CC | AA | TT | GG | CC/CC | MC1R*6/MC1R*6 |
| SP (1) | GG | CC | AA | TT | GG | **/** | MC1R*3/MC1R*3 |
| SP (2) | GG | CC | AA | TT | GG | **/** | MC1R*3/MC1R*3 |
| SP (3) | GG | CC | GA | TC | GA | **/** | MC1R*2/MC1R*3 |
| SP (4) | GG | CC | AA | TT | GG | **/** | MC1R*3/MC1R*3 |
| SP (5) | GG | CC | GA | TC | GA | **/** | MC1R*2/MC1R*3 |
| SP (6) | GG | CC | AA | TT | GG | **/** | MC1R*3/MC1R*3 |
| SP (7) | GG | CC | AA | TT | GG | **/** | MC1R*3/MC1R*3 |
| ZU (1) | GG | CC | AA | TT | GG | **/** | MC1R*3/MC1R*3 |
| ZU (2) | GG | CC | AA | TT | GG | **/** | MC1R*3/MC1R*3 |
| ZU (3) | GG | CC | AA | TT | GG | **/** | MC1R*3/MC1R*3 |
| ZU (4) | GG | CC | GA | TC | GA | **/** | MC1R*2/MC1R*3 |
| ZU (5) | GG | CC | GA | TC | GA | **/** | MC1R*2/MC1R*3 |
| ZU (6) | GG | CC | AA | TT | GG | **/** | MC1R*3/MC1R*3 |
| ZU (7) | GG | CC | GA | TC | AA | **/** | MC1R*2/- |

^1^ *MC1R* haplotypes: MC1R*2: GCGCA**; MC1R*3: GCATG**; and MC1R*6: GCATGCC
