# Supplementary material for: Identification of breed-specific genomic variants in Colombian Creole pig breeds by whole-genome sequencing
Source: Trop Anim Health Prod. 2023 Apr 11;55(3):154. doi: 10.1007/s11250-023-03557-9 (PMC10089996; doi:10.1007/s11250-023-03557-9)
Supplement: Supplementary file 3 — Supplementary Table S3 (DOCX 18.1 KB) [file 11250_2023_3557_MOESM3_ESM.docx]

**Supporting Table S3.** Individual genotypes of sequenced Colombian Creole pigs for the leptin receptor (*LEPR*) gene variants

| **Breed (pig no.)** | **Genotype ^1^** | | | | | | | | |
| --- | --- | --- | --- | --- | --- | --- | --- | --- | --- |
|  | **LEPR_2** | **LEPR_1** | **LEPR_3** | **LEPR_4** | **LEPR_5** | **LEPR_6** | **LEPR_7** | **LEPR_8** | **LEPR_9** |
| CM (1) | AGAAATAC/AGAAATAC | GG | AG | CC | TT | CT | GA | AT | GG |
| CM (2) | AGAAATAC/A | GG | AG | CT | TT | CC | GG | AA | AA |
| CM (3) | AGAAATAC/AGAAATAC | GA | AG | CC | TT | CC | GA | AT | AG |
| CM (4) | AGAAATAC/AGAAATAC | GA | AA | CC | TT | CT | GG | AA | AG |
| CM (5) | AGAAATAC/AGAAATAC | GA | GG | CC | TT | CC | GA | AT | AG |
| CM (6) | AGAAATAC/AGAAATAC | GG | GG | CC | TT | CC | AA | TT | GG |
| CM (7) | AGAAATAC/AGAAATAC | GG | AG | CC | TT | CT | GA | AT | GG |
| SP (1) | AGAAATAC/AGAAATAC | AA | AA | CC | TT | CC | GG | AA | AA |
| SP (2) | AGAAATAC/AGAAATAC | AA | AA | CC | TT | CC | GG | AA | AA |
| SP (3) | AGAAATAC/AGAAATAC | AA | AA | CC | TT | CC | GG | AA | AA |
| SP (4) | AGAAATAC/AGAAATAC | AA | AA | CC | TT | CC | GG | AA | AA |
| SP (5) | AGAAATAC/AGAAATAC | AA | AA | CC | TT | CC | GG | AA | AA |
| SP (6) | AGAAATAC/AGAAATAC | AA | AA | CC | TT | CC | GG | AA | AA |
| SP (7) | AGAAATAC/AGAAATAC | AA | AA | CC | TT | CC | GG | AA | AA |
| ZU (1) | AGAAATAC/A | GA | AA | CT | TT | CC | GG | AA | AA |
| ZU (2) | AGAAATAC/A | GG | AA | CT | TG | CC | GG | AA | AA |
| ZU (3) | AGAAATAC/AGAAATAC | AA | AA | TT | TT | CC | GG | AA | AA |
| ZU (4) | AGAAATAC/A | GG | AA | CT | TT | CC | GG | AA | AA |
| ZU (5) | AGAAATAC/A | GG | AA | CT | TG | CC | GG | AA | AG |
| ZU (6) | AGAAATAC/AGAAATAC | GA | AA | CC | TT | CC | GG | AA | AA |
| ZU (7) | AGAAATAC/AGAAATAC | GG | AA | CT | TG | CC | GG | AA | AG |

^1^ LEPR_2: g.146829573-146829580.AGAAATAC>A; LEPR_1: g.146829589.G>A; LEPR_3: g.146831558.A>G; LEPR_4: g.146838276.C>T; LEPR_5: g.146838380.T>G; LEPR_6: g.146847237.C>T; LEPR_7: g.146861093.G>A; LEPR_8: g.146861094.A>T; and LEPR_9: g.146861105.A>G.
